# Interoceptive sensibility predicts the ability to infer others’ emotional states

**DOI:** 10.1371/journal.pone.0258089

**Published:** 2021-10-06

**Authors:** Amelie M. Hübner, Ima Trempler, Corinna Gietmann, Ricarda I. Schubotz

**Affiliations:** 1 Department of Psychology, University of Muenster, Muenster, Germany; 2 Otto-Creutzfeldt-Center for Cognitive and Behavioural Neuroscience, University of Muenster, Muenster, Germany; Universite de Lille, FRANCE

## Abstract

Emotional sensations and inferring another’s emotional states have been suggested to depend on predictive models of the causes of bodily sensations, so-called interoceptive inferences. In this framework, higher sensibility for interoceptive changes (IS) reflects higher precision of interoceptive signals. The present study examined the link between IS and emotion recognition, testing whether individuals with higher IS recognize others’ emotions more easily and are more sensitive to learn from biased probabilities of emotional expressions. We recorded skin conductance responses (SCRs) from forty-six healthy volunteers performing a speeded-response task, which required them to indicate whether a neutral facial expression dynamically turned into a happy or fearful expression. Moreover, varying probabilities of emotional expressions by their block-wise base rate aimed to generate a bias for the more frequently encountered emotion. As a result, we found that individuals with higher IS showed lower thresholds for emotion recognition, reflected in decreased reaction times for emotional expressions especially of high intensity. Moreover, individuals with increased IS benefited more from a biased probability of an emotion, reflected in decreased reaction times for expected emotions. Lastly, weak evidence supporting a differential modulation of SCR by IS as a function of varying probabilities was found. Our results indicate that higher interoceptive sensibility facilitates the recognition of emotional changes and is accompanied by a more precise adaptation to emotion probabilities.

## 1 Introduction

Interoception, defined as the sense of the internal physiological state of the body [1], has gained growing interest in recent years because of its impact on physical and mental health, as well as on the processing of emotion. Classical appraisal theories of emotion processing postulate that emotional experiences to arise from the contextualised perception and interpretation of bodily responses to external stimuli [2–4]. The somatic marker hypothesis [5, 6] incorporated this view emphasizing viscerosensory origins of emotions [7]. This notion has been developed further under the framework of predictive coding, which presumes that emotional experiences are determined by inferences of the causes of bodily sensations based on past experiences. The interoceptive predictive coding model holds that emotional states are determined by the interplay of interoceptive predictions and interoceptive prediction errors [8–10]. Mismatches between descending interoceptive predictions and primary interoceptive afferents convey information about interoceptive changes and activate autonomous responses to restore physiological homeostasis or allostasis [9, 11]. Importantly, this interplay is flexibly tuned to the current reliability of exteroceptive and interoceptive signals by means of *precision*, regulating the relative weight accorded to prediction errors and predictions. Thus, highly precise prediction errors relative to prediction gives bias to bottom-up processing, whereas highly precise predictions relative to prediction errors give bias to top-down processing [12, 13].

Interoceptive precision may be a key to the striking differences individuals show in their interoceptive abilities. High sensitivity to interoceptive changes and, correspondingly, in emotional experience, is suggested to correspond with the ability to raise the precision of interoceptive prediction errors by focused attention [14]. As a result, in individuals with high versus low interoceptive sensitivity, interoceptive predictions are updated more frequently and thus become increasingly precise. The downside of this continual precision optimization can be observed in individuals with anxiety disorders, which promote increased attention to bodily signals [15]. In contrast, low interoceptive sensitivity has been shown to be accompanied by alexithymia, i.e., deficits in identifying and describing one’s own and others’ emotions [16, 17]. However, it should be noted that individuals could be differentiated on the basis of various measures of interoceptive abilities. Garfinkel and co-workers [18] distinguish between *interoceptive accuracy* (IAcc), operationalized as performance on objective detection of the heartbeat, *interoceptive sensibility* (IS) that quantifies the self-reported belief concerning one’s own perception of bodily signals, and *interoceptive awareness* (IAw), defined as a metacognitive measure of the correspondence between objective IAcc and subjective evaluation of one’s own interoception. Moreover, differences in interoceptive abilities could be associated with differences in physiological parameters that have been shown to reflect prediction error responses. For example, habituation through repeated exposure to a stimulus (i.e., decreased prediction error) is reflected in a decrease in skin conductance responses (SCRs) [19, 20]. In addition, SCRs have been shown to indicate preparatory or anticipatory reactions to upcoming events [21–23]. Thus, it is conceivable that individuals with a subjectively or objectively high interoceptive ability might also show stronger prediction error responses of the autonomic nervous system.

Individual differences in interoception could contribute to differences in social cognition. Studies provide evidence that the objective sensitivity for interoceptive changes, i.e., IAcc, relates to the ability to infer mental states of others (i.e., theory of mind) [24, 25]. Increased IAcc, as assessed by the heartbeat perception task, is related to an increased perceived arousal elicited by emotional stimuli [26, 27]. Moreover, individuals with higher IAcc are more sensitive to emotional facial expressions of others [28]. Consequently, the concept of interoceptive prediction has recently also augmented models of social cognition, i.e., inferring others’ intentions and emotional states based on exteroceptive, interoceptive and proprioceptive information. According to the concept of sensorimotor simulation, the sensorimotor system serves as a route for recognising facial expressions of emotion (see [29] for a review). During the observation of an emotional expression, we use our sensorimotor system to simulate the motor plan that the expresser is likely to use to produce the motor movements seen in the facial expression. This can be done explicitly, i.e. with facial mimicry, or without; crucially, the emotional meaning of the expression is inferred from our own prior exteroceptive and interoceptive experience of being in the presumed emotional state. Finally, the same principle can be applied to understanding others’ actions and mental states and how we share their bodily sensations [30]. For example, the predictions of one’s own interoceptive states that establish the sense of feeling of cold when one sees someone shivering. Consequently, emotion recognition benefits from adequate access to one’s own interoceptive cues. Thus, if individuals’ interoceptive abilities are reflected in their propensity to learn from prediction errors to acquire increasingly precise interoceptive predictive models, this would also make them better at inferring the emotional states of others.

The present study tested these assumptions by investigating the relationship between IS, assessed by the Multidimensional Assessment of Interoceptive Awareness, Version 2 (MAIA-2; [31]), and performance during a probabilistic emotion classification task with videotaped facial stimuli. We measured reaction times (RTs) along with SCRs as a widely used psychophysiological marker of changes in autonomic sympathetic arousal [32, 33] and activation to emotional stimuli [34, 35]. During the experiment, participants were required to indicate whether a neutral facial expression develops into a happy or fearful expression. Facial expressions at the end of the video varied in intensity to introduce different levels of uncertainty. Critically, we implemented different probabilities for the occurrence of either happy or fearful faces per block to assess participants’ propensity to efficiently update their predictive model. The varying probability and predictability of stimuli were quantified by information-theoretic measures, i.e., Shannon *surprise* and *entropy*, respectively [36]. The ‘surprise’ of an event, meaning its improbability, is given by the negative logarithm of the probability, whereas ‘entropy’ measures the average surprise of all possible events and quantifies the expected information of events regarding their predictability [37].

Regarding the discrimination of emotional change (hypothesis 1, H1), we expected that higher emotional intensity should lead to decreased RTs and increased SCRs reflecting decreased discrimination uncertainty [38, 39]. In addition, this effect should be more pronounced for fearful vs. happy facial expressions considering the consistent evidence for a superior recognition of happy faces compared to other facial expressions [40–42]. Therefore, we hypothesized that individuals with higher compared to lower IS would show decreased RTs and increased SCRs when discriminating emotions, especially when a fearful face of low intensity was presented.

Regarding the probabilistic context adaptation (hypothesis 2, H2), we expected that participants with higher vs. lower IS would learn the block-wise changing probabilistic imbalance more accurately due to stronger attentional precision-weighting [43]. This would be reflected in a positive correlation of surprise and entropy with RTs and SCRs in participants with higher IS but no correlation in participants with lower IS.

To explore whether the effects would be specific to emotion discrimination, we employed a non-emotional speeded classification control task, where participants were asked to discriminate the gender of a presented neutral face while facial stimuli developed from pixelated to high resolution. Paralleling the emotional condition, videos differed in resolution intensity (i.e., degree of pixilation) at the end of the video, with low resolution for low and high resolution for high intensity videos as well as in the probability for the occurrence of male or female faces.

## 2 Materials and methods

### 2.1 Participants

Forty-six right-handed healthy young volunteers (36 female, 10 male) with normal or corrected-to-normal vision were included in the present study. The participants’ age ranged from 18 to 32 years (22.9 ± 3.5 years). None of them reported a history of neurological or psychiatric disorders. The study protocol was conducted in accordance with ethical standards of the Declaration of Helsinki and approved by the Local Ethics Committee of the University of Muenster. Each participant submitted a signed informed consent form and received either reimbursement or course credits for their participation afterwards. Individuals provided written informed consent to permit for all potentially identifying information to be published.

### 2.2 Stimulus material

The stimulus material was created as part of a project on emotion recognition in patients with behavioural variant frontotemporal dementia in cooperation with the University Hospital Muenster, Germany [44]. The stimuli consisted of short videos with a mean duration of 3.00 s (± 0.39), which were displayed on a grey background. Videos depicted male or female faces posing from neutral to either happy or fearful emotional expressions (emotional condition) or male or female faces from pixelated to high resolution (non-emotional condition). Moreover, facial expressions in the emotional condition and image resolution in the non-emotional condition differed in terms of intensity (high/low) to introduce different levels of uncertainty. To control for potential effects of mouth opening on emotion recognition [45], fearful and happy expressions of high and low intensity were each presented in two different versions, i.e., with the mouth open and closed. Note that this factor was not considered in the statistical analyses to reduce the complexity of the statistical model.

To create these stimuli, we recorded several short video sequences (~ 2.08 to 4.40 s) of four actors and four actresses from four age groups each (20–30, 35–45, 50–60, 65–75 years). Actors and actresses were instructed to perform either a happy or a fearful facial expression of either high or low intensity and with mouth open or close. In order to achieve comparable length and development of the enfolding emotions, the subsequent videos were edited and cut using Adobe Premiere Pro CC (Adobe Systems Software, Dublin, Ireland) and Wondershare Filmora Version 8.5.1 (https://filmora.wondershare.com/). The tenth frame before the first (emotional) movement in the face was determined as a start frame which, thus, formed the neutral facial expression. The end frame was determined as the frame after which the emotion had reached its highest intensity and remained constant for another 20 frames. A total of 786 of these first and last frames were extracted and rated in two online-based pilot studies. In the first pilot study, a total of 60 participants (44 females; 27.4 ± 10.9; range 18–65 years) were asked to rate half of the start and end frames with regard to the valence and its intensity of the presented face on a 9-point scale ranging from 1 (*strong fear*), 5 (*neutral*) to 9 (*strong joy*) (see S1 File–containing all the supporting tables and figures). Moreover, if the participants experienced the emotion as neither fearful nor happy, they could enter a different emotion in a provided text field. In the second pilot study with a sample of 48 participants (33 females; 31.6 ± 14.8 years old; range 17–68 years), each participant rated valence, intensity and arousal of their subjective feelings elicited by the pictures on a scale ranging from 1 *(fear high / negative valence / calm*, *relaxed*), 5 (*neutral*) to 9 (*happy high / positive valence / exited*, *activated*). Based on the results of the pilot studies, we selected two videos per actor/actress for each of the eight conditions (happy vs. fearful x high vs. low intensity x mouth open vs. closed) that best met the main task’s requirements (i.e., neutral start, high/low intensity of happy/fearful expression in the end) resulting in 128 different videos in total. The final videos had a size of 800x800 pixels, a framerate of 25 frames per second, and an average length of 3.01 s. To create economically valid stimuli, we accepted that the emotional videos would slightly vary in length (*SD* = 0.39) (see S1 File). Therefore, we included video duration as a covariate in our analyses to control for possible confounds on the participants’ RT.

For the non-emotional condition, videos of each actor or actress with a constantly neutral expression were recorded. To ensure that all presented faces developed in the same time curve from pixelated to high resolution, they were provided with a Gaussian soft focus using Premiere Pro CC. The Gaussian soft focus was set to a value of 190 at the first frame, such that the faces were completely pixelated and not recognizable, as ensured by a further pilot study. From the fifth frame on, a linear decay was inducted. To introduce different levels of intensity comparable to the emotion task, the videos either dissolved completely or ran out with a remaining soft focus of 20 at the end frame. We additionally extended the grey background ellipsoid around the faces to ensure that noticeable features like the hairline would not favour ceiling effects. For each actor and actress, we created two videos with high and low intensity with a size of 1080x864 pixels, a framerate of 25 frames per second and an average length of 2.5 s.

### 2.3 Task

**Fig 1:**
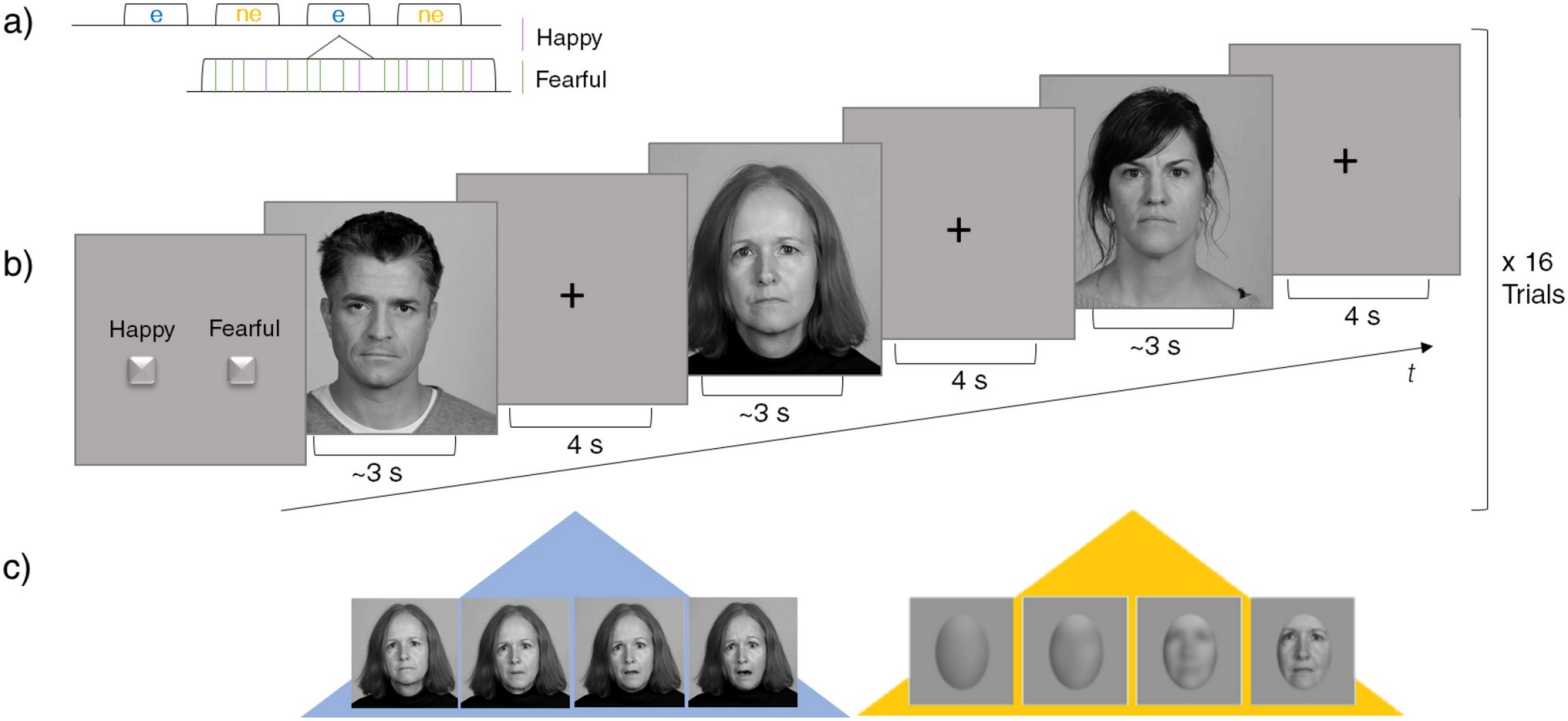
Schematic diagram of the task. **a)** Trials are presented in a mixed block- and event-related design with emotional (e) and non-emotional (ne) task blocks. **b)** At the beginning of each block, instructions indicate whether the participant has to indicate the emotion or the gender of the upcoming faces. One block includes 16 consecutive video trials. **c)** Videos depict actors/actresses posing from neutral to either happy or fearful facial expressions (blue background) or male or female actors/actresses from pixelated to high resolution (yellow background). The actor and actresses in the figure have given written informed consent (as outlined in PLOS consent form) to publish the photographs.

During the experiment, participants were seated in front of a computer screen located at a distance of about one meter. Videos were presented at the centre of the screen, separated by an interstimulus interval of 4 s during which a fixation cross was displayed centrally on the screen (Fig 1). The participants were asked to watch the presented videos attentively and to respond as fast and accurately as possible as soon as they recognized the emotion in the emotional condition or the gender of a presented face in the non-emotional condition. Participants responded by button press on a two-button response box, using their right-hand index and middle fingers. Stimulus-response mappings were counterbalanced across participants.

The task consisted of 32 emotional and non-emotional blocks with 16 consecutive videos each, i.e., a total of 512 trials (256 per condition). Thus, in the emotional condition each of the 128 videos was shown twice. Blocks were combined into four runs consisting of eight blocks each, i.e., after the reoccurrence of eight blocks of the same condition (emotional or non-emotional condition) the condition changed and remained the same for another eight blocks. At the beginning of each block, instructions were presented on the screen indicating whether the participant must respond to either the emotional expression or the gender of the faces. Each experimental block was followed by a break of 8 s, during which participants were given the information that the block had ended, followed by instructions regarding the upcoming block. Accordingly, the overall task for the emotional condition lasted about 44 min (2624 sec), for the control task about 39 min (2368 sec).

Within each block, either a high (75%) or low (25%) probability for the occurrence of fearful or happy faces in the emotional condition and either a high (75%) or low (25%) probability of male or female faces in the non-emotional condition were implemented. Stimuli were presented in a pseudo-randomized order ensuring that videos of one actor/actress were never repeated across consecutive trials. Transitions between the block types were balanced across the experiment.

Prior to the experiment, participants performed a short training session to get accustomed to the task. The training consisted of one block of 16 trials each and an equal probability for the different conditions.

The randomisation was programmed using MATLAB R2019b (The MathWorks Inc., Natick, MA, USA) and stimuli were presented using the Presentation software (Version 19.0, Neurobehavioral Systems, Inc., Berkeley, CA).

### 2.4 Assessment of interoceptive sensibility

For the self-assessment of interoceptive sensibility, the Multidimensional Assessment of Interoceptive Awareness, Version 2 (MAIA-2, [31]), a test that quantifies the self-reported belief concerning one’s own perception of bodily signals, was used. The MAIA-2 is a state-trait questionnaire with 37 items, which consist of eight subscales corresponding to its eight-factor structure: noticing (awareness of uncomfortable, comfortable, and neutral body sensations), not-distracting (tendency to ignore or distract oneself from sensations of pain or discomfort), not-worrying (emotional distress or worry with sensations of pain or discomfort), attention regulation (ability to sustain and control attention to body sensation), emotional awareness (awareness of the connection between body sensations and emotional states), self-regulation (ability to regulate psychological distress by attention to body sensations), body listening (actively listens to the body for insight), and trust (experiences one’s body as safe and trustworthy) [46]. Participants are asked to indicate on a 5-point Likert scale from 0 (*never*) to 5 (*always*) how often each statement applies to them in everyday life. Results of prior studies support the validity of the MAIA-2 scales, with Cronbach’s alpha for the eight scales ranging from 0.64 to 0.83 [31]. For our analyses, we calculated a total score per participant through reverse coding the corresponding items and summing all items. Together with other questionnaires assessing the participants’ emotion processing (see S1 File), the MAIA-2 was completed prior to the main task.

### 2.5 Skin conductance response acquisition

SCRs were acquired using the BrainVision Recorder Version 1.20.0801 (Brain Products, Munich, Germany). Two Ag/AgCl electrodes were placed on the annular and middle fingers of the participant’s left hand and 0.5%-NaCl electrode paste (GEL101; Biopac Systems) was used. Data were recorded at 500 Hz, using a sampling interval of 2000 μS. Preprocessing and data analysis were performed using PSPM [4.1.1], available at pspm.souorceforge.net. Skin conductance data were converted back to a waveform signal with 100 Hz time resolution, filtered with a unidirectional first-order Butterworth high pass filter with a cut-off frequency of 0.05 Hz, according to current recommendations [47]. Data were down-sampled to 10 Hz. The entire SCR time series was then *z*-transformed for each participant to account for interindividual differences in responsiveness [48]. The data were visually checked for artifacts, but no formal artifact rejection was implemented. The analysis of stimulus-locked (evoked) responses was done following the general linear convolution model (GLM) approach on a single-trial level. To this end, we extracted trial-by-trial estimates and ran the GLM with one regressor per trial. Each trial in the experiment was modelled as a Dirac delta function centred on the event onset, convolved with a canonical skin conductance response function (SCRF) and its first derivative [49]. From the estimated amplitude parameters for the canonical SCRF and its derivative, the response for each condition was reconstructed [47].

### 2.6 Data analysis

Basic statistical analyses were performed using R, version 3.6.2 [50]. For both behavioural and SCR data, false or missing responses were excluded from the analyses. Behavioural performance was defined by reaction times (RTs).

Comparisons of RTs and SCRs were carried out separately for the emotion and gender task. More specifically, we tested whether valence/gender, intensity and information-theoretic quantities, i.e., Shannon’s surprise *I(x*_*i*_*)* and entropy *H(X)* [36], reflecting the inverse probability and predictability of a single stimulus, respectively, could predict (variance in) RTs and SCRs on a single-trial level. While ‘surprise’ is a measure of the improbability of a particular event, ‘entropy’ measures the expected or average surprise over all events and thus reflects the predictability of an event within a particular context [37]. Shannon’s surprise was based on the frequency of a trial of a specific valence/gender *x*_*i*_ normalized by the sum of all past trials in the block:

$$p\left(x_{i}\right)=\frac{n\left(x_{i}\right)+1}{\sum x_{t}+1}$$

The prior counts before observing the first trial in the block were set to 1/2 for the two factor levels of valence (happy, fearful) and gender (male, female). The surprise *I(x*_*i*_*)* of each stimulus event given by the negative logarithm of this probability quantifies the amount of information provided by the current stimulus:

$$I\left(x_{i}\right)=-\text{ln}\;p(x_{i})$$

Finally, entropy *H*(*X*) measures the average surprise of all possible outcomes and quantifies the expected information of a stimulus regarding its predictability:

$$H\left(X\right)=\sum_{i}-p\left(x_{i}\right)\text{ln}\;p_{k}(x_{i})$$

We conducted generalized linear mixed-effects analyses using R, version 3.6.2 (R Core Team, 2019) via the package *lme4*, version 1.1.21 [51]. As the distribution of single-trial RTs was positively skewed, RTs were transformed to the natural logarithm to more closely approximate a normal distribution. Moreover, Q-Q plots indicated that residuals of RTs and SCR data were normally distributed. For the factors valence/gender and intensity, we used effect coding, with -1 for happy and 1 for fearful expressions, -1 for females and 1 for males, and -1 for high and 1 for low intensity expressions. Surprise and entropy were centered at individual levels, whereas MAIA-2 score was centered at the group level. Each model was fit with valence/gender and intensity (and their interaction), Shannon’s surprise and entropy and their respective interaction with the MAIA-2 score as fixed effects, and with a random intercept for each subject. For the emotional condition we added video duration to the models to control for effects of varying video length on RTs. Statistical significance for each fixed effect was calculated via *lmerTest*, version 3.1.1 [52], using the Satterthwaite’s approximation to denominator degrees of freedom. The significance level was set to α = .05. For posteriori pairwise comparisons we used *lsmeans* [53] with the Tukey adjustment for multiple tests and a high (25) and low (-25) level of the centred MAIA-2 score to assess differences between high and low IS participants.

In addition, we calculated Bayesian linear multilevel models in R [50] via the *brms* package and Stan using default priors [54, 55]. Regression coefficients and 95% credible intervals (CIs; i.e., Bayesian confidence intervals) are reported, meaning that the respective parameter falls within this interval with a 95% probability and indicating statistical significance on a 5% level if the interval does not contain zero.

## 3 Results

### 3.1 Emotional change recognition (H1)

#### 3.1.1 Behavioural data

**Fig 2:**
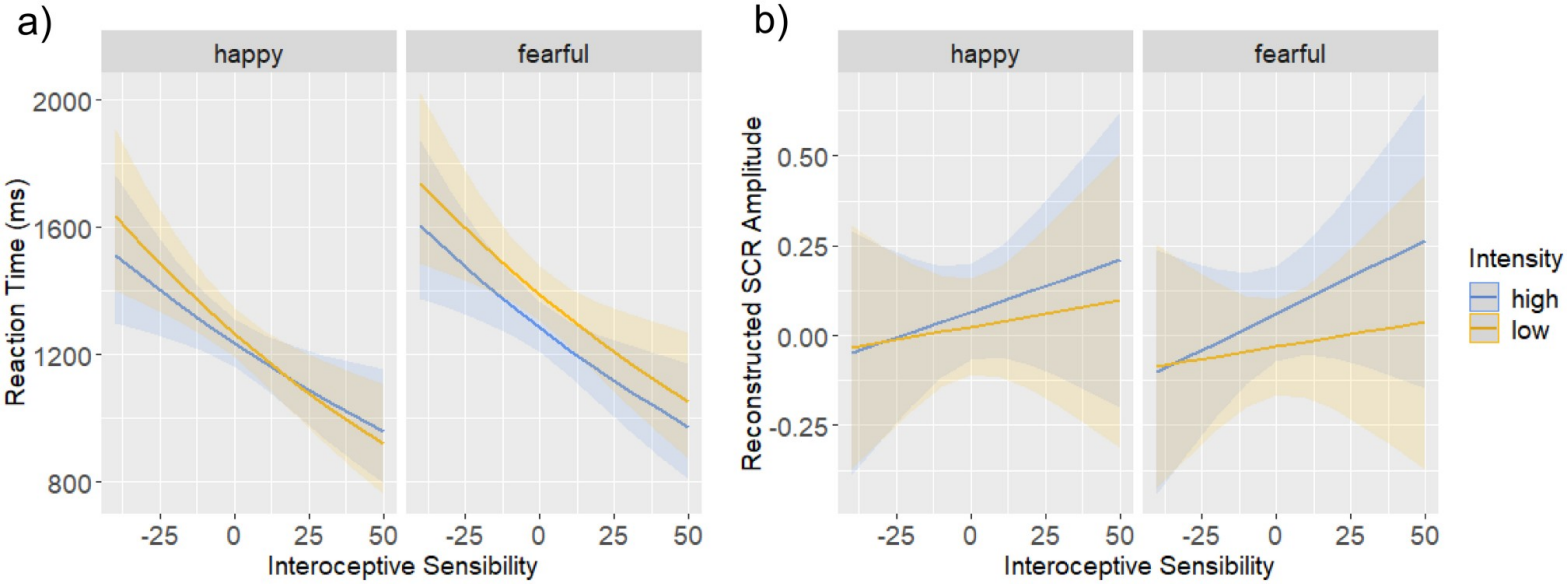
Effects of valence and intensity. Marginal effects of valence (happy, fearful) and intensity (high, low) as a function of interoceptive sensibility assessed by the MAIA-2 questionnaire (Mehling et al., 2018) on **a)** reaction time and **b)** skin conductance response (SCR). The solid lines depict the regression fit, and the shaded areas show the 95% confidence intervals.

Because the percentage of false alarms (2.27%) and missing data (0.14%) was low in the emotional condition, we restricted the behavioural analyses to RTs. The linear mixed-effect model predicting RTs revealed a significant main effect of IS, *b* = -0.01, *β* = -0.28, *t* = -3.16, *p* = 0.003. In line with our hypothesis, the negative gradient shows decreased RTs with increasing IS. We found a significant main effect of valence, *b* = 0.03, *β* = 0.10, *t* = 5.75, *p* < 0.001, with increased RTs for fearful facial expressions, and a main effect of intensity, *b* = 0.02, *β* = 0.07, *t* = 3.49, *p* < 0.001, driven by increased RTs for low intensity of an expression. Moreover, we found a significant interaction between valence and intensity, *b* = 0.01, *β* = 0.04, *t* = 5.54, *p* < 0.001, a significant interaction between intensity and IS, *b* = -0.001, *β* = -0.02, *t* = -2.37, *p* = 0.02, as well as a significant three-way interaction between valence, intensity and IS, *b* = 0.001, *β* = 0.02, *t* = 2.37, *p* = 0.02 (Fig 2a). Post-hoc tests comparing a low (-25) and a high (25) level of the centred MAIA-score revealed that, participants with lower IS showed increased RTs for high compared to low intensity of happy expressions, *b* = -0.06, *t* = -4.72, *p* < 0.001, and of fearful ones, *b* = -0.08, *t* = -6.59, *p* < 0.001. In contrast, participants with higher IS showed shorter RTs for high compared to low intensity of fearful expressions, *b* = -0.09, *t* = -6.42, *p* < 0.001, but no difference for happy expressions, *b* = 0.009, *t* = -0.74 *p* = 0.996. With decreasing IS, we found shorter RTs for happy compared to fearful expressions of both low, *b* = -0.07, *t* = -5.94, *p* < 0.001, and high intensity, *b* = -0.05, *t* = -4.27, *p* < 0.001. With increasing IS, RTs became shorter for happy compared to fearful expressions of low intensity, *b* = -0.11, *t* = -9.47, *p* < 0.001, but no difference when intensity was high, *b* = -0.03, *t* = -2.27, *p* = 0.313. In line with our hypothesis, participants with higher IS were thus faster in detecting emotional changes and slowed down their responses only to low intensity fearful expressions. Decreasing IS was accompanied by a gradual increase in RTs with increasing difficulty of the condition, i.e., from happy high to fearful low intensity expressions. In accordance with our assumptions, video length did not predict RTs, *b* = -0.007, *β* = -0.008, *t* = -1.11, *p* = 0.267.

The Bayesian logistic multilevel model on valence, intensity, surprise and entropy estimates, IS and their respective interactions, revealed significant main effects of valence, intensity and IS, as well as interaction effects of valence and intensity, intensity and IS, as well as a significant three-way interaction between valence, intensity and IS. A table with regression coefficients and corresponding 95% CIs for each variable predicting RTs in the emotional condition is given in the S1 File.

#### 3.1.2 SCR data

The linear mixed-effect model predicting SCRs revealed a trend for an effect of intensity corresponding to our hypothesis, *b* = -0.03, *β* = -0.02, *t* = -1.79, *p* = 0.07, driven by increased reconstructed SCR for high emotional expressions (Fig 2b). No main effect of valence (*p* = 0.41) or IS (*p* = 0.49) on SCR amplitudes, as well as no interaction effects were observed (all *p* > 0.34). The Bayesian logistic multilevel model predicting SCRs revealed no significant main or interaction effects.

### 3.2 Probabilistic context adaptation (H2)

#### 3.2.1 Behavioural data

**Fig 3:**
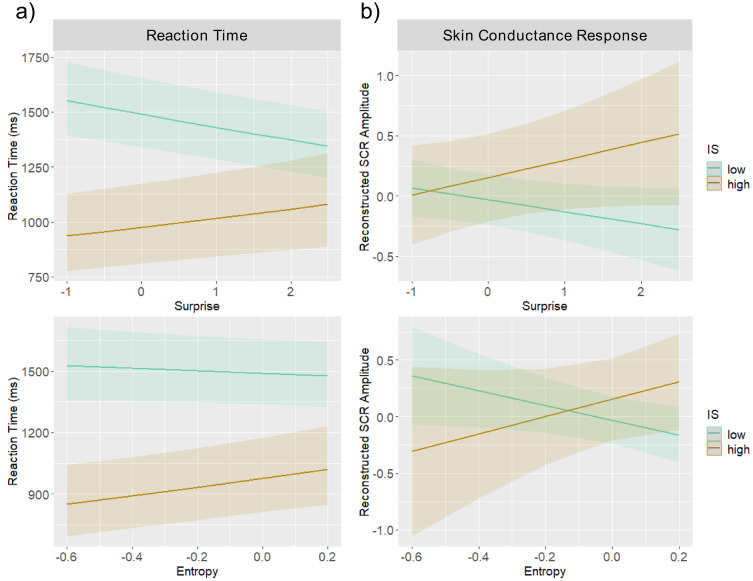
Effects of Shannon’s surprise and entropy. Results of the linear mixed effect regression model predicting **a)** reaction time **b)** and skin conductance response (SCR) by mean-centered surprise (top panel) and entropy (lower panel) as a function of interoceptive sensibility (IS) assessed by the MAIA-2 questionnaire [31]. For visualization purposes, marginal effects at representative values of the IS score (-25 for low, 25 for high IS) are presented. The solid lines depict the regression fit, and the shaded areas show the 95% confidence intervals.

We tested whether Shannon’s surprise and entropy of a single emotional expression in interaction with individual IS (i.e., continuously varying MAIA-2 scores) were predictive of the participants’ performance. The linear mixed-effect model predicting RTs revealed a significant main effect of surprise, *b* = -0.014, *β* = -0.02, *t* = -3.34, *p* < 0.001, and entropy, *b* = 0.059, *β* = 0.02, *t* = 2.10, *p* = 0.03, as well as significant interactions between IS and surprise, *b* = 0.001, *β* = 0.03, *t* = 4.58, *p* < 0.001, and between IS and entropy, *b* = 0.003, *β* = 0.02, *t* = 2.62, *p* = 0.01. Specifically, as expected in participants with higher IS surprise and entropy were positively correlated with increased RTs reflecting, in turn, a behavioral advantage in the course of learning from increasing probability of a specific valence. Contrary to our expectation, there also was a negative correlation in participants with lower IS (Fig 3a). The Bayesian logistic multilevel model revealed corresponding main effects of surprise and entropy, as well as interaction effects of surprise and entropy with IS (see S1 File).

#### 3.2.2 SCR data

In the linear mixed-effect model predicting SCRs, we observed weak evidence for our hypothesis suggesting an interaction effect between IS and surprise, *b* = 0.003, *β* = 0.02, *t* = 1.78, *p* = 0.07, and between IS and entropy, *b* = 0.019, *β* = 0.02, *t* = 1.79, *p* = 0.07, but no main effects of surprise (*p* = 0.572) and entropy (*p* = 0.316) (Fig 3b). Higher IS, thus, appeared to be accompanied by increased SCRs to unpredicted and unpredictable valences. However, the Bayesian logistic multilevel model did not capture these effects and did not reveal main or interaction effects.

### 3.3 Control task

#### 3.3.1 Behavioural data

In the non-emotion control condition, i.e., the gender task, the percentage of false alarms (5.17%) and missing data (0.78%) was significantly higher than in the emotional condition, although still relatively low (*M* = 0.07, *SD* = 0.05, *t*(45) = 6.54, *p* < 0.001). The linear mixed regression model for the prediction of RTs revealed a significant main effect of gender, *b* = 0.016, *β* = 0.05, *t* = 5.53, *p* < 0.001, driven by increased RTs for female faces compared to male faces. As expected, the main effect of intensity was also significant, *b* = -0.031, *β* = -0.09, *t* = -10.25, *p* < 0.001, with decreased RTs for faces presented with high resolution compared to low resolution. In contrast to the emotional condition, the interaction between gender and intensity was not significant (*p* = 0.62) and there was no interaction effect between gender, intensity and IS (*p* = 0.76) (see S1 File). Likewise, the Bayesian logistic multilevel model also revealed a main effect of gender *b* = 0.02, 95%-CI [0.01, 0.02] and intensity *b* = -0.03, 95%-CI [-0.04, 0.03].

However, contrary to our expectation the linear mixed regression model as well as the Bayesian logistic multilevel model for the prediction of RTs revealed a significant main effect of entropy, *b* = 0.14, *β* = 0.04, *t* = 4.13, *p* < 0.001, as well as a trend for a significant interaction between surprise and IS, *b* = 0.00, *β* = -0.02, *t* = 1.90, *p* = 0.057. No other main or interaction effects were significant (all *p* ≥ 0.48).

#### 3.3.2 SCR data

The linear mixed effect model on reconstructed SCR amplitudes did not reveal significant main effects of gender (*p* = 0.88), intensity (*p* = 0.53) or interaction effects with IS (*p* ≥ 0.29). Likewise, the linear mixed effect model did not reveal significant main effects with entropy and surprise or interaction effects with entropy (all *p* ≥ 0.19). Nevertheless, we found an interaction effect of surprise and IS, *b* = 0.004, *β* = 0.02, *t* = 2.15, *p* = 0.03. Likewise, the Bayesian logistic multilevel model predicting SCRs in the non-emotional control condition revealed no significant main or interaction effects, except the interaction effect of surprise and IS, *b* = 0.00, 95%-CI [0.00, 0.01].

## 4 Discussion

The present study tested whether an individual’s interoceptive sensibility (IS) is positively related to the speed of recognizing emotional changes in others’ facial expressions and to the ability to exploit biased probabilities to adapt expectations of facial emotions. We found (1) decreased RTs in individuals with increasing IS. Individuals with decreased IS were slower in recognizing low vs. high emotional intensity and fearful vs. happy valence of facial expressions. Individuals with higher IS slowed down their responses only for the most difficult condition, i.e., when a fearful face of low intensity was presented. Regarding differences in the exploitation of biased probabilities of happy vs. fearful faces, (2) participants with higher IS were faster in recognizing more probable (i.e., less surprising) and more predictable (i.e., less uncertain) facial emotions, whereas participants with lower IS displayed lower RTs for more probable as well as more predictable facial emotions. A trend for corresponding effects of surprise and entropy were also observed for the SCRs. Finally, we found mixed evidence for the hypothesis that (3) interindividual differences in IS particularly impact emotion but not gender expectation and recognition. Although IS had no impact on RTs or SCRs in the gender discrimination task, the IS score tended to interact with gender surprise similar as with emotion surprise.

### 4.1 Interoceptive sensibility accompany lower thresholds for emotional change recognition

Our behavioral results confirmed the hypothesis that self-reported sensibility to one’s own interoceptive states (*interoceptive sensibility*, IS) modulates the recognition of emotional changes in facial expressions of others. Specifically, while individuals with increased IS showed decreased RTs only for high compared to low intensity of fearful expressions, lower IS participants showed a graduation of performance depending on both valence and intensity by becoming slower at recognizing fearful vs. happy and low vs. high intensity expressions.

While our study is the first to show a relationship between IS and emotion recognition, previous studies also suggested that interoception influences the sensitivity to others’ emotions. Terasawa et al. [28] previously reported that individuals with high interoceptive accuracy (IAcc), assessed by the heartbeat detection task, show lower thresholds for the detection of various emotions in facial expressions, especially of happy ones. In addition, individuals with higher sensitivity to visceral changes experience emotional stimuli as more arousing [27, 56]. Our results extend these findings by showing that when tested for emotion recognition speed, individuals with lower IS benefit more from high intensities of facial emotions than individuals with higher IS. Considering that in daily life our spontaneous facial expressions are mostly of low to intermediate intensity, whereas high intensity is an exception [57–59], individuals with low IS conceivably display more difficulties in inferring emotional states from others’ facial expressions in their daily social interaction.

Regarding the valence of facial expressions, participants with increased IS recognized fearfulness equally as well as happiness when intensity was high, whereas individuals with lower IS showed a general advantage for the recognition of happy faces. Previous studies found that happy faces are recognized faster and more accurately than other types of facial expressions [40–42], even when presented at low intensity [60, 61]. Theoretical explanations for this effect include that the expression of happiness requires simpler physical changes and occurs more frequently in our daily life [42, 62, 63]. On the contrary, although fearful facial expressions are *detected* fast e.g. [64, 65], they are less well *recognized* than other facial expressions [10, 62, 65–67]. In the present study, the participants’ task was to discriminate between happy and fearful expressions such that RT measures reflected recognition rather than detection of the emotional expression. The ability to perceive one’s own bodily signals has been shown to lead to enhanced emotional discernment in social processing by promoting empathic abilities as well as emotional expressions [68–70]. Against this backdrop, we take the discrimination advantage of higher IS in our sample to be particularly evident for those stimuli that are especially difficult to be unequivocally discriminated, that is, fearful in comparison to happy faces and emotional expressions of low vs. high intensity.

As for the SCRs, although we expected an increased amplitude for fearful vs. happy expressions as SCRs are assumed to reflect physiological arousal to highly activating stimuli which are to be detected fast [34, 35], our findings did not provide evidence for this assumption. A possible explanation could be the lateralisation of the SCR profiles of the hands in response to emotional facial stimuli. Banks et al. [71] found stronger SCRs to anger, disgust and fear on the right hand, while higher amplitudes were found for the left hand in responses to sad, happy and neutral faces. Thus, possible differences in SCR amplitudes in response to fearful vs. happy faces could have been influenced by the SCR recordings from the left hand in our study. Alternatively, the difference between emotion recognition and detection could also provide an explanation for why no SCR difference was found between the two conditions: while emotion detection is associated with high arousal, for which SCR is a highly sensitive measure, emotion recognition implies cognitive appraisal, which possibly involves only subtle changes in SCR. Thus, as increases in SCRs are induced by multiple emotional states, it was found not to be a very specific measure of different emotions [72]. In line with this, there was weak evidence to support our hypothesis that high vs. low expressions (i.e., higher arousal) would elicit stronger SCRs, as has been observed in previous studies [73].

However, we neither found general differences between participants with high vs. low IS in SCR amplitude nor that higher IS facilitates sympathetic arousal in certain experimental conditions. Although previous EEG studies reported increased amplitudes for both the P300 component and slow waves in response to emotional pictures in individuals with high vs. low interoceptive sensibility [27, 56], other studies did not find overall differences in SCR varying with interoceptive sensibility [74]. Our findings provide no evidence for a significant correlation between the IS score or emotion processing and autonomous arousal. As discussed in the following, SCR patterns could be rather characterized by increased attention to these signals.

### 4.2 Interoceptive sensibility facilitates context-sensitive emotional inference

Using a novel task with videos showing neutral faces, developing to emotional expressions and occurring with different probabilities, the present results confirmed our hypothesis that higher IS is accompanied by a more precise adaptation of emotional predictions. RTs of individuals with higher IS increased when the neutral facial expression unfolded into a rather unexpected, i.e., surprising emotion (as measures by the current probability of a specific emotion occurrence), and decreased with increasing predictability, i.e., lower entropy of an emotion occurrence. In contrast, low IS individuals showed the opposite effects, with decreased RTs to surprising and unpredictable emotions. This correlational pattern suggests that higher IS individuals benefited from the implemented probabilistic context more than individuals with lower IS.

According to Ainley et al. [43], an attentional mechanism is at the basis of higher IS individuals’ enhanced precision of prediction errors. Jiang and co-workers [75] report that attention during a face-scene discrimination task, i.e., independent of emotion processing, accelerates prediction error processing as reflected in an increased neural patterns classifier’s performance in distinguishing between expected and unexpected signals. Moreover, using EEG, Petzschner et al. [76] find that interoceptive attention modulates the cortical processing of heartbeats, as suggested by increased heartbeat-evoked potentials. Attention might, thus, manifest as increased precision of the heartbeat or other context-specific ascending interoceptive signals, and reduced precision of currently irrelevant interoceptive signals. This precision-weighting would ultimately lead to more precise interoceptive predictions. Accordingly, expected vs. unexpected negative vs. neutral facial expressions lead to decreased heartbeat-evoked potentials [77]. Furthermore, better perception of visceral cues facilitates unaware conditioned responding as well as the prediction of shocks [78, 79].

Against this background our findings further show, probably due to stronger attentional precision-weighting, individuals with higher IS learn better from, and hence build stronger expectations based on biased probabilities of emotional expressions. This in turn leads to a faster recognition of predicted emotions, but also to hesitation when unexpected emotions occur. In contrast, individuals with decreased IS show the opposite pattern by responding slower to predictable but faster to surprising stimuli. Moreover, although the effects on SCRs were generally weak and should be interpreted with caution, the (non-significant) trend for a modulation of SCRs by surprise and entropy in interaction with IS paralleled this RT pattern, with surprise and entropy positively covarying with SCRs in higher IS individuals but negatively in lower IS individuals. It was previously suggested that SCRs reflect an orienting reflex in response to novel or unexpected stimuli [19, 80]. Thus, the increase of SCRs as a function of entropy and surprise in higher IS individuals is in accordance with our hypotheses, reflecting adaptation to biased probabilities and heightened arousal in response to violations. However, the effect found in low IS individuals was rather unexpected. As a suggestion, our findings could be interpreted along the lines of a dual processing mode [81] assuming a supervisory attentional system [82]: In individuals with higher IS, expected emotions may be processed more automatically, whereas the classification of rather unexpected emotions requires increased cognitive control. As for the opposite effects in individuals with low IS, the emotional classification processes could generally be less controlled, which would be reflected in high RTs and SCRs even for frequently occurring emotions. Thus, the present results provide evidence that differences in IS regarding context-sensitive emotional inference are reflected in differences in bodily responses to emotional stimuli that vary in probability of their occurrence, although the concrete processes underlying this relationship must be clarified in future studies.

Finally, it has been suggested that learning of emotion concepts and affective predictions depending on past experience (i.e., memory), affected by various environmental influences (i.e., stability and habitual patterns of selective attention), determine whether sensory input is experienced as emotional or not [10, 83]. Consequently, prediction might even change how we perceive neutral stimuli [84, 85]. Since we used dynamic video-taped stimuli starting with a neutral facial expression evolving into an emotional expression, it is possible that individuals with higher IS already perceived neutral facial expressions as more emotional according to the respective emotional context, which led to a recognition advantage when prediction was fulfilled. To validate this interpretation, future studies could examine whether interindividual differences in IS determine the influence of affective predictions on the perception of neutral stimuli.

### 4.3 Domain specific and domain unspecific effects of interoceptive sensibility

Since attention is suggested to modulate precision-weighting of prediction errors in general [75] and also represents a key construct in various subscales of the MAIA-2 questionnaire [31], effects of IS on emotion discrimination could be simply due to an improved ability to focus attention on any type of sensory input rather than being specifically caused by differences in interoceptive abilities. We therefore implemented a non-emotional control task where participants had to indicate the gender of neutral faces in videos developing from highly pixelated to high resolution in order to assess detection of changes independent of observed bodily cues [30]. The recognition of gender did not differ between these individuals, that is, no evidence was found for a general discrimination advantage in high vs. low IS individuals. However, regarding the probabilistic manipulation generating an expectation bias for female or male faces, surprise and IS tended to interact, albeit not significantly, such that RTs increased as a function of surprise more in high than in low IS individuals. This effect suggests a subtle impact of prediction errors on behavior in higher IS individuals which is independent of the prediction domain.

Our finding is in accordance with the postulation of at least three different areas of predictive functioning, i.e., the exteroceptive, interoceptive, and proprioceptive dimension, underlying perception, emotion, and action, respectively [9]. These can operate both amodal and multimodal, depending on the level of the predictive hierarchy at which predictions are violated. It is conceivable that in the present study, prediction errors in the exteroceptive modality (i.e., in the gender task) also elicited bodily signals on which higher IS individuals direct their focus more than lower IS individuals. As to the emotion task, the interaction was much more pronounced and an additional interaction effect with entropy was observed. Although our findings suggest partially domain-spanning interindividual differences in predictive performance, interoceptive sensitivity and information appear to play a greater role for the recognition of emotion than gender.

### 4.4 Strengths and limitations

Although the present study is the first investigating whether higher IS comes with better recognition and expectation of both emotional and non-emotional stimuli, the evidence for domain-specific and domain-general effects of IS has to be further validated in future studies. For instance, in order to determine interindividual differences in interoceptive abilities, we used a self-report questionnaire, i.e., the MAIA-2, assuming that different dimensions of interception are correctly tapped by subjective assessment. As several studies report an independence of IS and IAcc as an objective measure of interoceptive abilities [18, 86–89], future approaches could additionally use IAcc to examine the influence of interoceptive abilities on emotion discrimination.

Moreover, we acknowledge the relatively small sample size that plays a role especially with regard to the investigation of interindividual variation. Given that only trends could be observed regarding the effects of SCR, these results should be interpreted with caution. However, our study is particularly characterized by the fact that we created dynamically enfolding emotional facial expressions to investigate the recognition of emotional changes as well as the impact of differences in IS in an economically valid way. Only recently, the need to study dynamic and real situational emotions (as opposed to static Emoji-like expressions) was emphasized in order to get a valid picture of how emotional meaning is inferred [90].

Since social factors play a striking role in the development and maintenance of mental illness [91], our findings provide further evidence that interoception is an important feature of diagnosis and treatment of different psychiatric disorders [92]. Consequently, approaches to improve interoceptive abilities are worth discussing. As such, a therapeutic approach called mindful awareness in body-oriented therapy (MABT) could be appointed, specifically designed to teach skills of interoceptive awareness through a combination of psychoeducation and somatic approaches [93]. Future studies could investigate whether approaches to improve interoception also positively influence social cognition.

## 5 Conclusion

The present study suggests that interoceptive sensibility facilitates the speed of recognition of emotional changes in facial expressions of others, potentially mediated by an increased attention to bodily signals. Furthermore, higher interoceptive sensibility entails a more precise adaptation to biased probabilities of emotional valences, pointing to a stronger reliance on situationally adjusted prediction. Correspondingly, bodily responses tend to increase for less probable emotions. Future studies can build on these findings by assessing corresponding effects in clinical populations associated with interoceptive dysfunctions such as anxiety disorders or alexithymia.

## Supporting information

S1 File (DOCX)

## References

[1] Craig A. D. (2002). How do you feel? Interoception: the sense of the physiological condition of the body. *Nature reviews neuroscience*, 3(8), 655. doi: 10.1038/nrn894 12154366

[2] Schachter S., & Singer J. (1962). Cognitive, social, and physiological determinants of emotional state. *Psychological Review*, 69(5), 379. 14497895 10.1037/h0046234

[3] James W. (1884). What is an Emotion?(188–205). *Mind*, 9, 34.

[4] Lange C. G. (1885). The mechanism of the emotions. *The Classical Psychologists*, 672–684.

[5] Bechara A., Damasio H., & Damasio A. R. (2000). Emotion, decision making and the orbitofrontal cortex. *Cerebral Cortex*, 10(3), 295–307. doi: 10.1093/cercor/10.3.295 10731224

[6] Damasio A. R. (1996). The somatic marker hypothesis and the possible functions of the prefrontal cortex. *Philosophical Transactions of the Royal Society of London*. *Series B*: *Biological Sciences*, 351(1346), 1413–1420. doi: 10.1098/rstb.1996.0125 8941953

[7] Damasio A. R. (2010). Self Comes to Mind: Constructing the Conscious Brain. London: Vintage Books.

[8] Seth A. K. (2013). Interoceptive inference, emotion, and the embodied self. *Trends in Cognitive Sciences*, 17(11), 565–573. doi: 10.1016/j.tics.2013.09.007 24126130

[9] Seth A. K., & Friston K. J. (2016). Active interoceptive inference and the emotional brain. *Philosophical Transactions of the Royal Society B*: *Biological Sciences*, 371(1708). doi: 10.1098/rstb.2016.0002 28080966 PMC5062097

[10] Smith R., Parr T., & Friston K. J. (2019). Simulating Emotions: An Active Inference Model of Emotional State Inference and Emotion Concept Learning. *Frontiers in Psychology*, 10(December), 1–24. doi: 10.3389/fpsyg.2019.02844 31920873 PMC6931387

[11] Seth A. K., Suzuki K., & Critchley H. D. (2012). An interoceptive predictive coding model of conscious presence. *Frontiers in Psychology*, 3(JAN), 1–16. doi: 10.3389/fpsyg.2011.00395 22291673 PMC3254200

[12] Friston K. (2008). Hierarchical models in the brain. *PLoS Computational Biology*, 4(11). 10.1371/journal.pcbi.1000211 PMC2570625 18989391

[13] Friston K. (2010). The free-energy principle: A unified brain theory? *Nature Reviews Neuroscience*, 11(2), 127–138. doi: 10.1038/nrn2787 20068583

[14] Ainley V., Apps M. A. J., Fotopoulou A., & Tsakiris M. (2016). “Bodily precision”: A predictive coding account of individual differences in interoceptive accuracy. *Philosophical Transactions of the Royal Society B*: *Biological Sciences*, 371(1708). 10.1098/rstb.2016.0003 PMC5062093 28080962

[15] Domschke K., Stevens S., Pfleiderer B., & Gerlach A. L. (2010). Interoceptive sensitivity in anxiety and anxiety disorders: an overview and integration of neurobiological findings. *Clinical Psychology Review*, 30(1), 1–11. doi: 10.1016/j.cpr.2009.08.008 19751958

[16] Herbert B. M., Herbert C., & Pollatos O. (2011). On the relationship between interoceptive awareness and alexithymia: is interoceptive awareness related to emotional awareness? *Journal of Personality*, 79(5), 1149–1175. doi: 10.1111/j.1467-6494.2011.00717.x 21241306

[17] Brewer R., Cook R., & Bird G. (2016). Alexithymia: A general deficit of interoception. *Royal Society Open Science*, 3(10). doi: 10.1098/rsos.150664 27853532 PMC5098957

[18] Garfinkel S. N., Seth A. K., Barrett A. B., Suzuki K., & Critchley H. D. (2015). Knowing your own heart: Distinguishing interoceptive accuracy from interoceptive awareness. *Biological Psychology*, 104, 65–74. doi: 10.1016/j.biopsycho.2014.11.004 25451381

[19] Geuter S., Boll S., Eippert F., & Büchel C. (2017). Functional dissociation of stimulus intensity encoding and predictive coding of pain in the insula. ELife, 6, 1–22. doi: 10.7554/eLife.24770 28524817 PMC5470871

[20] Barry R. J. (2009). Habituation of the orienting reflex and the development of preliminary process theory. *Neurobiology of learning and memory*, 92(2), 235–242. doi: 10.1016/j.nlm.2008.07.007 18675927

[21] Bach D. R., Flandin G., Friston K. J., & Dolan R. J. (2010). Modelling event-related skin conductance responses. *International Journal of Psychophysiology*, 75(3), 349–356. doi: 10.1016/j.ijpsycho.2010.01.005 20093150 PMC2877881

[22] Delgado M. R., Nearing K. I., LeDoux J. E., & Phelps E. A. (2008). Neural circuitry underlying the regulation of conditioned fear and its relation to extinction. Neuron, 59(5), 829–838. doi: 10.1016/j.neuron.2008.06.029 18786365 PMC3061554

[23] Marschner A., Kalisch R., Vervliet B., Vansteenwegen D., & Büchel C. (2008). Dissociable roles for the hippocampus and the amygdala in human cued versus context fear conditioning. *Journal of Neuroscience*, 28(36), 9030–9036. doi: 10.1523/JNEUROSCI.1651-08.2008 18768697 PMC6670865

[24] Sowden S., Brewer R., Catmur C., & Bird G. (2016). The specificity of the link between alexithymia, interoception, and imitation. *Journal of Experimental Psychology*: *Human Perception and Performance*, 42(11), 1687–1692. doi: 10.1037/xhp0000310 27786535 PMC5082312

[25] Shah P., Catmur C., & Bird G. (2017). From heart to mind: Linking interoception, emotion, and theory of mind. *Cortex; a Journal Devoted to the Study of the Nervous System and Behavior*, 93, 220. 28476292 10.1016/j.cortex.2017.02.010 PMC5542037

[26] Dunn B. D., Galton H. C., Morgan R., Evans D., Oliver C., Meyer M., et al. (2010). Listening to your heart: How interoception shapes emotion experience and intuitive decision making. Psychological Science, 21(12), 1835–1844. doi: 10.1177/0956797610389191 21106893

[27] Pollatos O., Kirsch W., & Schandry R. (2005). On the relationship between interoceptive awareness, emotional experience, and brain processes. *Cognitive Brain Research*, 25(3), 948–962. doi: 10.1016/j.cogbrainres.2005.09.019 16298111

[28] Terasawa Y., Moriguchi Y., Tochizawa S., & Umeda S. (2014). Interoceptive sensitivity predicts sensitivity to the emotions of others. *Cognition and Emotion*, 28(8), 1435–1448. doi: 10.1080/02699931.2014.888988 24559130

[29] Wood A., Rychlowska M., Korb S., & Niedenthal P. (2016). Fashioning the face: sensorimotor simulation contributes to facial expression recognition. *Trends in cognitive sciences*, 20(3), 227–240. doi: 10.1016/j.tics.2015.12.010 26876363

[30] Ondobaka S., Kilner J., & Friston K. (2017). The role of interoceptive inference in theory of mind. Brain and Cognition, 112, 64–68. doi: 10.1016/j.bandc.2015.08.002 26275633 PMC5312780

[31] Mehling W. E., Acree M., Stewart A., Silas J., & Jones A. (2018). The multidimensional assessment of interoceptive awareness, version 2 (MAIA-2). PLoS ONE, 13(12), 1–12. doi: 10.1371/journal.pone.0208034 PMC6279042 30513087

[32] Critchley H. D. (2002). Electrodermal responses: What happens in the brain. *Neuroscientist*, 8(2), 132–142. doi: 10.1177/107385840200800209 11954558

[33] Kleckner I. R., Zhang J., Touroutoglou A., Chanes L., Xia C., Simmons W. K., et al. (2017). Evidence for a large-scale brain system supporting allostasis and interoception in humans. *Nature Human Behaviour*, 1(5), 69. doi: 10.1038/s41562-017-0069 28983518 PMC5624222

[34] Bradley M. M., Codispoti M., Cuthbert B. N., & Lang P. J. (2001). Emotion and motivation I: defensive and appetitive reactions in picture processing. *Emotion*, 1(3), 276. 12934687

[35] Bernat E., Patrick C. J., Benning S. D., & Tellegen A. (2006). Effects of picture content and intensity on affective physiological response. *Psychophysiology*, 43(1), 93–103. doi: 10.1111/j.1469-8986.2006.00380.x 16629689 PMC2242429

[36] Shannon C. E. (1948). A mathematical theory of communication. *Bell System Technical Journal*, 27(3), 379–423.

[37] Strange B. A., Duggins A., Penny W., Dolan R. J., & Friston K. J. (2005). Information theory, novelty and hippocampal responses: unpredicted or unpredictable?. *Neural Networks*, 18(3), 225–230. doi: 10.1016/j.neunet.2004.12.004 15896570

[38] Calder A. J., Young A. W., Rowland D., & Perrett D. I. (1997). Computer-enhanced emotion in facial expressions. *Proceedings of the Royal Society of London*. *Series B*: *Biological Sciences*, 264(1383), 919–925. doi: 10.1098/rspb.1997.0127 9265191 PMC1688436

[39] Matsumoto D., & Hwang H. S. (2011). Judgments of facial expressions of emotion in profile. *Emotion*, 11(5), 1223. doi: 10.1037/a0024356 21942701

[40] Ekman P. (1982). Does the face provide accurate information? *Emotion in the Fuman Face*.

[41] Kirita T., & Endo M. (1995). Happy face advantage in recognizing facial expressions. *Acta Psychologica*, 89(2), 149–163.

[42] Leppänen J. M., & Hietanen J. K. (2003). Affect and Face Perception: Odors Modulate the Recognition Advantage of Happy Faces. *Emotion*, 3(4), 315–326. doi: 10.1037/1528-3542.3.4.315 14674826

[43] Ainley V., Apps M. A. J., Fotopoulou A., & Tsakiris M. (2016). “Bodily precision”: A predictive coding account of individual differences in interoceptive accuracy. *Philosophical Transactions of the Royal Society B*: *Biological Sciences*, 371(1708). 10.1098/rstb.2016.0003 PMC5062093 28080962

[44] Hübner, A. M., Trempler, I., Kloth, N., Duning, T., Johnen, A., Schubotz, R. I. (2021). Selective Impairment of Emotion Inference in Behavioural Variant Frontotemporal Dementia? A Pilot Study. Manuscript in preparation.

[45] Langeslag S. J., Gootjes L., & van Strien J. W. (2018). The effect of mouth opening in emotional faces on subjective experience and the early posterior negativity amplitude. *Brain and cognition*, 127, 51–59. doi: 10.1016/j.bandc.2018.10.003 30316954

[46] Mehling W. E., Price C., Daubenmier J. J., Acree M., Bartmess E., & Stewart A. (2012). The Multidimensional Assessment of Interoceptive Awareness (MAIA). *PloS one*, 7(11), e48230. doi: 10.1371/journal.pone.0048230 23133619 PMC3486814

[47] Bach D. R., Friston K. J., & Dolan R. J. (2013). An improved algorithm for model-based analysis of evoked skin conductance responses. *Biological Psychology*, 94(3), 490–497. doi: 10.1016/j.biopsycho.2013.09.010 24063955 PMC3853620

[48] Bach D. R., Flandin G., Friston K. J., & Dolan R. J. (2009). Time-series analysis for rapid event-related skin conductance responses. *Journal of Neuroscience Methods*, 184(2), 224–234. doi: 10.1016/j.jneumeth.2009.08.005 19686778 PMC2772899

[49] Bach D. R., Daunizeau J., Friston K. J., & Dolan R. J. (2010). Dynamic causal modelling of anticipatory skin conductance responses. *Biological psychology*, 85(1), 163–170. doi: 10.1016/j.biopsycho.2010.06.007 20599582 PMC2923733

[50] R Core Team. (2019). A Language and Environment for Statistical Computing. R Foundation for Statistical Computing, Vienna, Austria. URL http://www.R-project.org/.

[51] Bates D., Maechler M., Bolker B., Walker S., Christensen R. H. B., Singmann H., et al. (2015). Package ‘lme4’. *Convergence*, 12(1), 2.

[52] Kuznetsova A., Brockhoff P. B., & Christensen R. H. (2017). lmerTest package: tests in linear mixed effects models. Journal of statistical software, 82(1), 1–26.

[53] Lenth R. V. (2016). Least-squares means: the R package lsmeans. *Journal of statistical software*, 69(1), 1–33.

[54] Bürkner P.-C. (2017). brms: An R Package for Bayesian Multilevel Models Using Stan. *Journal of Statistical Software*, 80(1), 1–28. doi: 10.18637/jss.v080.i01

[55] Carpenter B., Gelman A., Hoffman M. D., Lee D., Goodrich B., Betancourt M., et al. (2017). Stan: A Probabilistic Programming Language. *Journal of Statistical Software*, 76(1). doi: 10.18637/jss.v076.i01 PMC9788645 36568334

[56] Herbert B. M., Pollatos O., & Schandry R. (2007). Interoceptive sensitivity and emotion processing: An EEG study. *International Journal of Psychophysiology*, 65(3), 214–227. doi: 10.1016/j.ijpsycho.2007.04.007 17543405

[57] Matsumoto D., & Hwang H. C. (2014). Judgments of subtle facial expressions of emotion. Emotion, 14(2), 349. doi: 10.1037/a0035237 24708508

[58] Hess U., Adams R. B., Grammer K., & Kleck R. E. (2009). Face gender and emotion expression: Are angry women more like men? *Journal of Vision*, 9(12), 19. doi: 10.1167/9.12.19 20053110

[59] Motley M. T., & Camden C. T. (1988). Facial expression of emotion: A comparison of posed expressions versus spontaneous expressions in an interpersonal communication setting. *Western Journal of Communication (Includes Communication Reports)*, 52(1), 1–22.

[60] Hess U., Blairy S., & Kleck R. E. (1997). The intensity of emotional facial expressions and decoding accuracy. *Journal of Nonverbal Behavior*, 21(4), 241–257. doi: 10.1023/A:1024952730333

[61] Palermo R. (2004). SpringerLink—Behavior Research Methods, Volume 36, Number 4. *Behavior Research Methods*, 36(4), 634–638. doi: 10.3758/BF03206544 15641409

[62] Adolphs R. (2002). Recognizing emotion from facial expressions: psychological and neurological mechanisms. *Behavioral and Cognitive Neuroscience Reviews*, 1(1), 21–62. doi: 10.1177/1534582302001001003 17715585

[63] Montagne B., Kessels R. P. C., De Haan E. H. F., & Perrett D. I. (2007). The emotion recognition task: A paradigm to measure the perception of facial emotional expressions at different intensities. *Perceptual and Motor Skills*, 104(2), 589–598. doi: 10.2466/pms.104.2.589-598 17566449

[64] Bayet L., Quinn P. C., Laboissière R., Caldara R., Lee K., & Pascalis O. (2017). Fearful but not happy expressions boost face detection in human infants. *Proceedings of the Royal Society B*: *Biological Sciences*, 284(1862). doi: 10.1098/rspb.2017.1054 PMC5597829 28878060

[65] Smith F. W., & Rossit S. (2018). Identifying and detecting facial expressions of emotion in peripheral vision. *PLoS ONE*, 13(5), 1–15. doi: 10.1371/journal.pone.0197160 29847562 PMC5976168

[66] Palermo R., & Coltheart M. (2004). Photographs of facial expression: Accuracy, response times, and ratings of intensity. *Behavior Research Methods*, *Instruments*, *& Computers*, 36(4), 634–638. 10.3758/bf03206544 15641409

[67] Rapcsak S. Z., Galper S. R., Comer J. F., Reminger S. L., Nielsen L., Kaszniak A. W., et al. (2000). Fear recognition deficits after focal brain damage: a cautionary note. *Neurology*, 54(3), 575. doi: 10.1212/wnl.54.3.575 10680785

[68] Arnold A. J., Winkielman P., & Dobkins K. (2019). Interoception and Social Connection. *Frontiers in Psychology*, 10, 1–6. 31849741 10.3389/fpsyg.2019.02589 PMC6901918

[69] Decety J. (2011). Dissecting the neural mechanisms mediating empathy. *Emotion Review*, 3(1), 92–108.

[70] Zaki J. (2014). Empathy: a motivated account. *Psychological Bulletin*, 140(6), 1608. doi: 10.1037/a0037679 25347133

[71] Banks S. J., Bellerose J., Douglas D., & Jones-Gotman M. (2012). Bilateral skin conductance responses to emotional faces. *Applied psychophysiology and biofeedback*, 37(3), 145–152. doi: 10.1007/s10484-011-9177-7 22407530

[72] Kreibig S. D. (2010). Autonomic nervous system activity in emotion: A review. *Biological psychology*, 84(3), 394–421. doi: 10.1016/j.biopsycho.2010.03.010 20371374

[73] Fusar-Poli P., Landi P., & O’Connor C. (2009). Neurophysiological response to emotional faces with increasing intensity of fear: A skin conductance response study. *Journal of Clinical Neuroscience*, 16(7), 981–982. doi: 10.1016/j.jocn.2008.09.022 19362481

[74] Werner N. S., Kerschreiter R., Kindermann N. K., & Duschek S. (2013). Interoceptive awareness as a moderator of affective responses to social exclusion. *Journal of Psychophysiology*, 27(1), 39–50. doi: 10.1027/0269-8803/a000086

[75] Jiang J., Summerfield C., & Egner T. (2013). Attention sharpens the distinction between expected and unexpected percepts in the visual brain. *Journal of Neuroscience*, 33(47), 18438–18447. doi: 10.1523/JNEUROSCI.3308-13.2013 24259568 PMC3834051

[76] Petzschner F. H., Weber L. A., Wellstein K. V., Paolini G., Do C. T., & Stephan K. E. (2019). Focus of attention modulates the heartbeat evoked potential. *NeuroImage*, 186, 595–606. doi: 10.1016/j.neuroimage.2018.11.037 30472370

[77] Gentsch A., Sel A., Marshall A. C., & Schütz‐Bosbach S. (2019). Affective interoceptive inference: Evidence from heart-beat evoked brain potentials. *Human Brain Mapping*, 40(1), 20–33. doi: 10.1002/hbm.24352 30159945 PMC6865546

[78] Katkin E. S., Wiens S., & Öhman A. (2001). Nonconscious fear conditioning, visceral perception, and the development of gut feelings. *Psychological Science*, 12(5), 366–370. doi: 10.1111/1467-9280.00368 11554668

[79] Raes A. K., & De Raedt R. (2011). Interoceptive awareness and unaware fear conditioning: Are subliminal conditioning effects influenced by the manipulation of visceral self-perception? *Consciousness and Cognition*, 20(4), 1393–1402. doi: 10.1016/j.concog.2011.05.009 21684179

[80] Bradley M. M. (2009). Natural selective attention: Orienting and emotion. *Psychophysiology*, 46(1), 1–11. doi: 10.1111/j.1469-8986.2008.00702.x 18778317 PMC3645482

[81] Schneider W., & Chein J. M. (2003). Controlled & automatic processing: behavior, theory, and biological mechanisms. *Cognitive science*, 27(3), 525–559.

[82] Norman, D. A., & Shallice, T. (1980). Attention to Action: Willed and Automatic Control of Behavior. Technical Report No. 8006.

[83] Barrett L. F. (2017). The theory of constructed emotion: an active inference account of interoception and categorization. *Social Cognitive and Affective Neuroscience*, 12(1), 1–23. doi: 10.1093/scan/nsw154 27798257 PMC5390700

[84] Anderson E., Siegel E., White D., & Barrett L. F. (2012). Out of sight but not out of mind: Unseen affective faces influence evaluations and social impressions. *Emotion*, 12(6), 1210. doi: 10.1037/a0027514 22506501 PMC4957816

[85] Siegel E. H., Wormwood J. B., Quigley K. S., & Barrett L. F. (2018). Seeing what you feel: Affect drives visual perception of structurally neutral faces. *Psychological science*, 29(4), 496–503. doi: 10.1177/0956797617741718 29485945 PMC5902425

[86] Calì G., Ambrosini E., Picconi L., Mehling W. E., & Committeri G. (2015). Investigating the relationship between interoceptive accuracy, interoceptive awareness, and emotional susceptibility. *Frontiers in Psychology*, 6, 1–13. 26379571 10.3389/fpsyg.2015.01202 PMC4547010

[87] Garfinkel S. N., & Critchley H. D. (2013). Interoception, emotion and brain: new insights link internal physiology to social behaviour. Commentary on:: “Anterior insular cortex mediates bodily sensibility and social anxiety” by Terasawa et al. (2012). *Social Cognitive and Affective Neuroscience*, 8(3), 231–234. doi: 10.1093/scan/nss140 23482658 PMC3594730

[88] Bornemann B., Herbert B. M., Mehling W. E., & Singer T. (2014). Differential changes in self-reported aspects of interoceptive awareness through 3 months of contemplative training. *Frontiers in Psychology*, 5(OCT), 1–13. doi: 10.3389/fpsyg.2014.01504 25610410 PMC4284997

[89] Farb N., Daubenmier J., Price C. J., Gard T., Kerr C., Dunn B. D., et al. (2015). Interoception, contemplative practice, and health. *Frontiers in Psychology*, 6(JUN), 1–26. doi: 10.3389/fpsyg.2015.00763 26106345 PMC4460802

[90] Barrett L. F., Adolphs R., Marsella S., Martinez A. M., & Pollak S. D. (2019). Emotional expressions reconsidered: Challenges to inferring emotion from human facial movements. *Psychological science in the public interest*, 20(1), 1–68. doi: 10.1177/1529100619832930 31313636 PMC6640856

[91] Silva M., Loureiro A., & Cardoso G. (2016). Social determinants of mental health: a review of the evidence. *The European Journal of Psychiatry*, 30(4), 259–292.

[92] Khalsa S. S., & Lapidus R. C. (2016). Can interoception improve the pragmatic search for biomarkers in psychiatry?. *Frontiers in psychiatry*, 7, 121. doi: 10.3389/fpsyt.2016.00121 27504098 PMC4958623

[93] Price C. J., & Hooven C. (2018). Interoceptive awareness skills for emotion regulation: Theory and approach of mindful awareness in body-oriented therapy (MABT). *Frontiers in psychology*, 9, 798. doi: 10.3389/fpsyg.2018.00798 29892247 PMC5985305

