# Supplementary material for: Interoceptive sensibility predicts the ability to infer others’ emotional states
Source: PLoS One. 2021 Oct 6;16(10):e0258089. doi: 10.1371/journal.pone.0258089 (PMC8494315; doi:10.1371/journal.pone.0258089)
Supplement: S1 File — (DOCX) [file pone.0258089.s001.docx]

**Supplementary Material for:**

Interoceptive sensibility predicts the ability to infer others’

emotional states

Amelie M. Hübner^1*^, Ima Trempler^1,2*^, Corinna Gietmann^1^, Ricarda I. Schubotz^1,2^

^1^ Department of Psychology, University of Muenster, Germany

^2^ Otto-Creutzfeldt-Center for Cognitive and Behavioural Neuroscience, University of Muenster, Germany

* Authors contributed equally to the manuscript

*1. Stimulus Material*

In the first pilot study a total of 60 participants (44 females; 27.4 ± 10.9; range 18-65 years) were asked to rate half of the start and end frames with regard to the depicted valence and its intensity on a 9-point scale ranging from 1 (*strong fear*), 5 (*neutral*) to 9 (*strong joy*). We recoded the variable so that we had one variable for the happy expressions with values ranging from 0 (*neutral*) to 4 (*strong joy*) and one for the fearful expressions with values ranging from 0 (*neutral*) to 4 (*strong fear*). We calculated two-way ANOVAs on the rating of the end and start frames of the final set of selected videos including the factors valence and intensity. For the end frames, there was a main effect of intensity, *F*(1, 124) = 164.41, *p* < 0.001, but no main effect of valence, *p* = 0.904, and no interaction of valence and intensity, *p* = 0.795. No differences between the conditions were found for the start frames (all *p* ≥ .185) (Figure S1). Moreover, the corresponding ANOVA was calculated with video duration as dependent variable. We found a small but significant effect of intensity, *F*(1, 124) = 5.84, *p* = 0.017, but no effect of valence and no interaction effect between the two factors (*p* ≥ .182) (Figure S2).


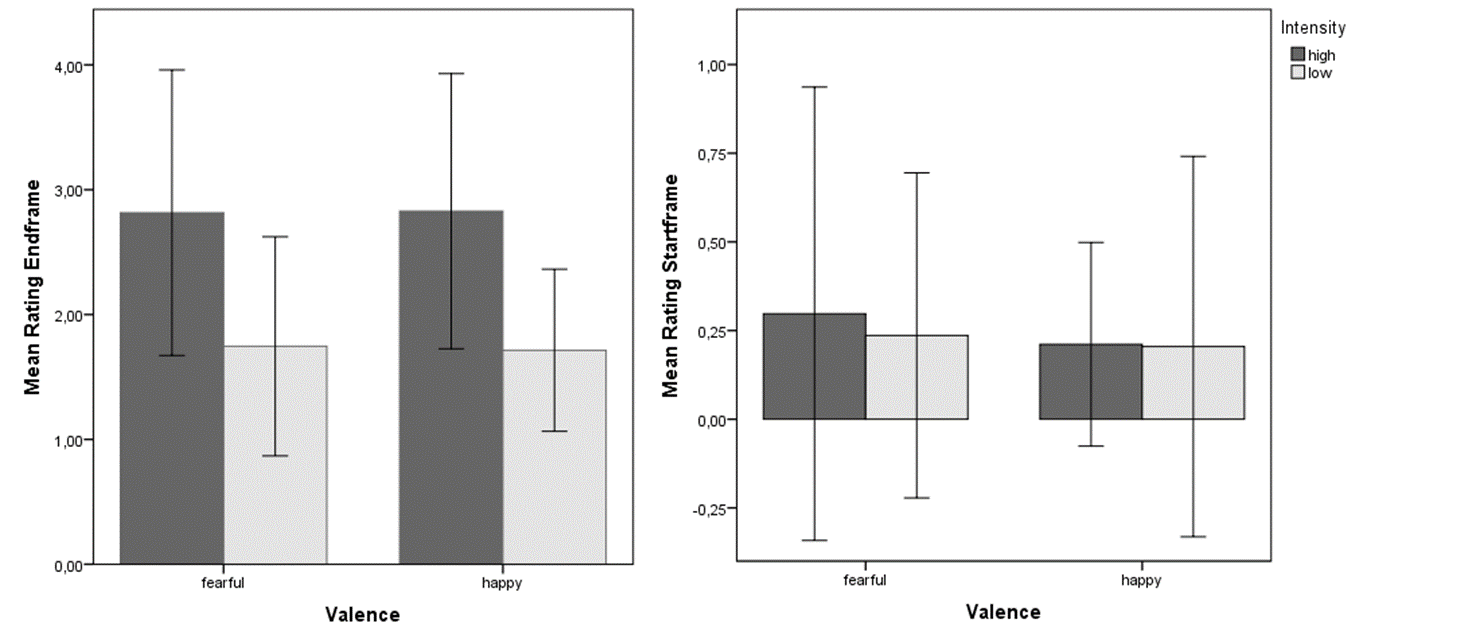
Figure S1. Mean intensity rating for the two conditions of the end and start frame of the final video set. Error bars represent two standard deviations from the mean.


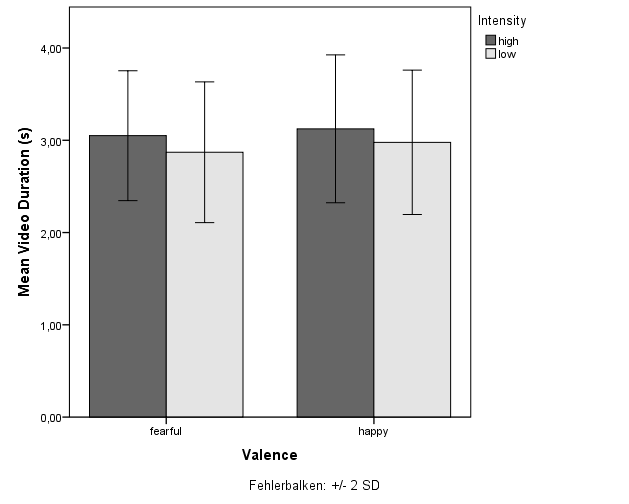


Figure S2. Mean duration of the final video set depending on condition. Error bars represent two standard deviations from the mean.

*2. Additional Questionnaires*

In addition to the MAIA-2, the questionnaire of self-assessment of emotional competencies (SEK-27) (Berking & Znoj, 2008) was completed, which includes nine scales with 27 items, three for each scale: attention, body awareness, clarity, understanding, regulation, acceptance, tolerance resilience, self-support, willingness to confront. Based on the close link between interoception and empathy and the assumption that a precise prediction of internal states also implies increased empathy for others (Ernst, Northoff, Böker, Seifritz, & Grimm, 2013; Ondobaka et al., 2017), we also added the Saarbruecken personality questionnaire in the self-report version (SPF) (Paulus, 2009). The SPF is a german version of the Interpersonal Reactivity Index (IRI) by Davis (1983), consisting of 16 items covering a total of four scales: empathic concern, fantasy, personal distress, assumption of perspectives. We also added the short form of the Trait Emotional Intelligence Questionnaire (TEIQue-SF). This is a 30-item questionnaire designed to measure global trait emotional intelligence (trait EI) based on the long form of the TEIQue (Furnham & Petrides, 2003).

To explore the relationship between the questionnaires and RTs, we calculated Pearson’s correlation coefficients separately for the performance in the emotion and gender task. For the correlation of RTs with the MAIA-2, we found significant negative correlations for the emotion task (*r* = -0.429, *p* = 0.002) as well as for the gender task (*r* = -0.299, *p* = 0.044). For the SPF, we found significant negative correlations for the emotion task (*r* = -0.379, *p* = 0.01) and the gender task (*r* = -0.296, *p* = 0.046). The correlation between the mean RT per participant and the SEK did not reach significance neither in the emotion task (*r* = -0.250, *p* = 0.094) nor in the gender task (*r* = -0.124, *p* = 0.41). Also, there were no significant correlations of TEI-score with RTs in the emotion task (*r* = -0.078, *p* = 0.61) or the gender task (*r* = 0.066, *p* = 0.66).

*3. Results of the Bayesian logistic multilevel model predicting RTs*

Table S1. Bayesian logistic multilevel model predicting RTs in the emotional condition

| Coefficient | *b* | l-95%  CI | u-95%  CI |
| --- | --- | --- | --- |
| Video duration | -0.01 | -0.02 | 0.01 |
| Valence | 0.03 | 0.03 | 0.04 |
| Intensity | 0.03 | 0.02 | 0.03 |
| IS | -0.01 | -0.01 | -0.00 |
| Surprise | -0.01 | -0.02 | -0.01 |
| Entropy | 0.05 | 0.00 | 0.09 |
| Valence x Intensity | 0.01 | 0.01 | 0.02 |
| Valence x IS | 0.00 | -0.00 | 0.00 |
| Intensity x IS | -0.00 | -0.00 | -0.00 |
| Surprise x IS | 0.00 | 0.00 | 0.00 |
| Entropy x IS | 0.00 | 0.00 | 0.01 |
| Valence x Intensity x IS | 0.00 | 0.00 | 0.00 |

*4. Control Task*


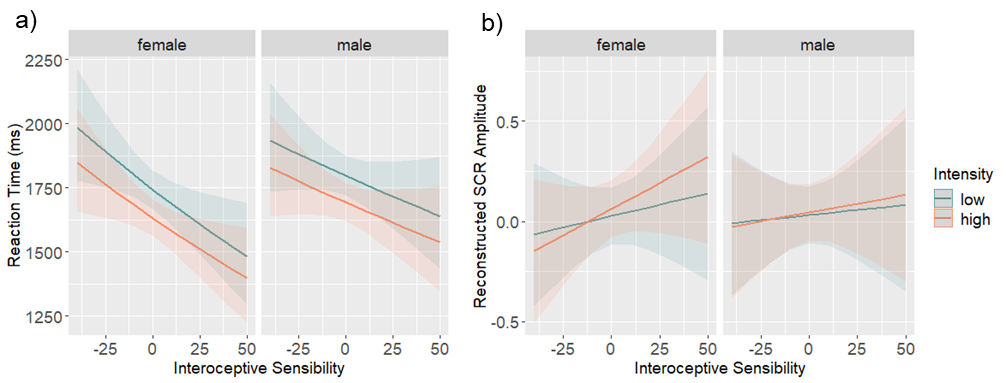


**Figure S3**. Marginal effects of gender (female, male) and intensity (high, low) as a function of interoceptive sensibility on **a)** reaction time and **b)** skin conductance response (SCR). The solid lines depict the regression fit, and the shaded areas show the 95% confidence intervals.

References

Berking, M., & Znoj, H. (2008). Entwicklung und Validierung eines Fragebogens zur standardisierten Selbsteinschätzung emotionaler Kompetenzen (SEK-27). Zeitschrift für Psychiatrie, Psychologie und Psychotherapie, 56(2), 141-153.

Davis, M. H. (1983). Measuring individual differences in empathy: Evidence for a multidimensional approach. *Journal of personality and social psychology*, *44*(1), 113.

Ernst, J., Northoff, G., Böker, H., Seifritz, E., & Grimm, S. (2013). Interoceptive awareness enhances neural activity during empathy. Human Brain Mapping, 34(7), 1615-1624.

Furnham, A., & Petrides, K. V. (2003). Trait emotional intelligence and happiness. Social Behavior and Personality: an international journal, 31(8), 815-823.

Ondobaka, S., Kilner, J., & Friston, K. (2017). The role of interoceptive inference in theory of mind. Brain and cognition, 112, 64-68.

Paulus, C. (2009). Der Saarbrücker Persönlichkeitsfragebogen SPF (IRI) zur messung von empathie: psychometrische evaluation der deutschen version des interpersonal reactivity index.
